# Comment on “Effect of Riociguat and Sildenafil on Right Heart Remodeling and Function in Pressure Overload Induced Model of Pulmonary Arterial Banding”

**DOI:** 10.1155/2018/6593682

**Published:** 2018-08-19

**Authors:** Asger Andersen

**Affiliations:** Aarhus University Hospital, Dep. Cardiology, Denmark

It was a pleasure to read the paper “Effect of Riociguat and Sildenafil on Right Heart Remodeling and Function in Pressure Overload Induced Model of Pulmonary Arterial Banding” by Rai and colleagues [1] in the recent issue of BioMed Research International. It is an important topic and by using magnetic resonance imaging (MRI) to characterize right ventricular (RV) performance it adds to previous studies on the same subject [2–6]. The authors conclude that both sildenafil and riociguat prevent RV failure in mice subjected to pulmonary artery banding (PAB) based on improvement in stroke volume (SV) and ejection fraction (EF). It does however seem that there is a miscalculation in the EF and SV parameters reported by the authors. By using the parameters reported by the authors to recalculate, SV is reduced and the improvement in EF is less pronounced in riociguat treated animals which would change the interpretation of the results. Stroke volume was calculated based on MRI derived parameters as SV=EDV-ESV and EF was calculated as EF=SV/EDV. EDV is reported as 79.6 *μ*l (placebo), 59.9 *μ*l (sildenafil), and 44.0 *μ*l (riociguat). ESV is reported as 54.7 *μ*l (placebo), 33.9 *μ*l (sildenafil), and 24.7 *μ*l (riociguat). This would yield an EF of 31% (placebo), 43% (sildenafil), and 44% (riociguat) and a SV of 24.9 *μ*l (placebo), 26 *μ*l (sildenafil), and 19.3 *μ*l (riociguat). The authors report EF of 30% (placebo), 45% (sildenafil), and 58% (riociguat) and a SV of 24.2 (placebo), 25.9 (sildenafil), and 28.5 *μ*l (riociguat). This discrepancy should be clarified, as it could potentially change the interpretation and the conclusion of the present study.

Another comment is that previous studies with a similar design in a rat PAB model [6] found that mortality was increased with the soluble guanylate cyclase (sGC) stimulator BAY 41-2272 (a compound similar to riociguat). It should be reported in the paper by Rai and colleagues whether there was any mortality in the treatment arms in this study, as this could potentially introduce a selection bias.

I hope that the authors will address the comments above to improve the knowledge on sGC-cGMP stimulation in RV failure.
